# Association of Acute Respiratory Infections with Indoor Air Pollution from Biomass Fuel Exposure among Under-Five Children in Jimma Town, Southwestern Ethiopia

**DOI:** 10.1155/2021/7112548

**Published:** 2021-12-23

**Authors:** Abebaw Addisu, Tesfalem Getahun, Mulunesh Deti, Yilkal Negesse, Besufekad Mekonnen

**Affiliations:** ^1^Department of Public Health, College of Medicine and Health Science, Mizan–Tepi University, Mizan, Ethiopia; ^2^Department of Environmental Health, College of Public Health, Jimma University, Jimma, Ethiopia; ^3^Department of Epidemiology, College of Medicine and Health Science, Mizan–Tepi University, Mizan, Ethiopia

## Abstract

**Background:**

Most of the households in developing countries burn biomass fuel in traditional stoves with incomplete combustion that leads to high indoor air pollution and acute respiratory infections. Acute respiratory infection is the most common cause of under-five morbidity and mortality accounting for 2 million deaths worldwide and responsible for 18% of deaths among under-five children in Ethiopia. Although studies were done on acute respiratory infections, the majority of studies neither clinically diagnose respiratory infections nor use instant measurement of particulate matter.

**Methods:**

The community-based cross-sectional study design was employed among under-five children in Jimma town from May 21 to June 7, 2020. A total of 265 children through systematic random sampling were included in the study. The data were collected using a pretested semistructured questionnaire and laser pm 2.5 meter for indoor particulate matter concentration. Associations among factors were assessed through correlation analysis, and binary logistic regression was done to predict childhood acute respiratory infections. Variables with *p*-value less than 0.25 in bivariate regression were the candidate for the final multivariate logistic regression. Two independent sample *t*-tests were done to compare significant mean difference between concentrations of particulate matter.

**Results:**

Among 265 under-five children who were involved in the study, 179 (67.5%) were living in households that predominantly use biomass fuel. Prevalence of acute respiratory infections in the study area was 16%. Children living in households that use biomass fuel were four times more likely to develop acute respiratory infections than their counterparts (AOR: 4.348; 95% CI: 1.632, 11.580). The size of household was significantly associated with the prevalence of acute respiratory infections. Under-five children living in households that have a family size of six and greater had odds of 1.7 increased risk of developing acute respiratory infections than their counterparts (AOR: 1.7; 95% CI: 1.299, 2.212). The other factor associated with acute respiratory infection was separate kitchen; children living in households in which there were no separate kitchen were four times at increased risk of developing acute respiratory infection than children living in households which have separate kitchen (AOR: 4.591; 95% CI: 1.849, 11.402). The concentration of indoor particulate matter was higher in households using biomass fuel than clean fuel. There was statistically higher particulate matter concentration in the kitchen than living rooms (*t* = 4.509, *p* ≤ 0.001). Particulate matter 2.5 concentrations (*μ*g/m^3^) of the households that had parental smoking were significantly higher than their counterparts (AOR: 20.224; 95% CI: 1.72, 12.58).

**Conclusion:**

There is an association between acute respiratory infections and biomass fuel usage among under-five children. Focusing on improved energy sources is essential to reduce the burden and assure the safety of children.

## 1. Introduction

Household air pollution can result from fuels that are used for cooking, indoor tobacco smoking, insecticides and pest controls, and building materials and chemicals used for cleaning purposes [1]. Incomplete combustion of biomass fuel for domestic energy requirement with very traditional stoves in three-rock adjustment leads to high indoor air pollution [2]. Globally, about 2.4 billion people rely on biomass fuel as their main source of domestic energy and most of those people live in developing countries, where more than 90% of people cook using biomass fuel [3]. Unlike developed countries, with near universal electrification, households in developing countries often choose solid fuels like animal dung, wood, crop residue, and charcoal over clean fuels [4].

In sub-Saharan Africa, 77% of energy needs of households are met by burning biomass fuels, mainly for household cooking and heating [5]. Nearly 99% of rural communities and 80% of urban dwellers in Ethiopia primarily use solid fuel for cooking and heating. The overall biomass fuel consumption in the country is about 95% [6].

Exposures to biomass fuels are associated with many respiratory diseases such as acute respiratory infections, chronic obstructive pulmonary disease, lung cancer, pulmonary tuberculosis, and asthma [7]. Exposure to biomass fuel smoke from a traditional stove is one of the factors leading to acute respiratory infections among under-five children [8]. Acute respiratory infection is the most common causes of under-five illness and mortality that account for 94,037,000 disability adjusted life years and 2 million deaths worldwide [1]. In developing countries, exposure to biomass fuel combustion has increased incidence of acute respiratory infections in children below age of five years [9]. Acute respiratory infection is responsible for 50,320 annual deaths of children under five years, accounting for 4.9% national burden of disease in Ethiopia [10]. Although several studies were done in Ethiopia on acute respiratory infections (ARI) and biomass fuel use, previous studies were only limited on establishing the association between biomass fuel exposure and occurrence of respiratory infections. The majority of studies neither clinically diagnose the respiratory infections nor use instant measurement of particulate matter. Thus, the purpose of this study was to determine the magnitude of acute respiratory infections and its association with exposure to biomass fuel and measure the indoor concentration of suspended particulate matter at household level.

## 2. Methods

### 2.1. Study Design and Setting

Community based cross-sectional study design was conducted to examine the association of acute respiratory infections with biomass fuel exposure among under-five children in Jimma town, from May 21 to June 7, 2020. The study was conducted in Jimma town, which is the capital of Jimma zone. It is one of the towns in Oromia regional state located in the Southwestern Ethiopia at 352 Km from the capital of Ethiopia, Addis Ababa. The town has 17 Kebeles containing total population of about 128,306 among which 8915 are under-five children. It is found at an altitude of about 1,780 m above sea level. It is generally characterized by warm climate with a mean annual temperature range from maximum 25–30°C and minimum 7–20°C and annual rain fall range from 1200 to 2000 mm. The maximum precipitation occurs during the three months from June to August [11].

### 2.2. Sample Size and Sampling Procedure

The sample size was calculated using a single population proportion formula using the following parameters: 95% confidence level (1.96) and 5% margin of error (0.05); the prevalence of children with acute respiratory infection from previous studies with comparable study design at Sidama was taken as 21% [12] and 5% nonrespondent rate. *n* = [Z^2^*p* (1 − p)/w^2^] [13]. As a result, total sample size was 265 under-five children. Children under the age of five years in Jimma town were the source population from which the study participants were taken and selected children in each Kebele were engaged in the study. Households were taken as the sampling unit and the mothers or caregivers of the children were the study unit in the sampling process. Jimma town has 17 villages/Kebeles, which are small administrative units. The town has a total of 8915 under five children. For the sake of representation and small geographic area of the study, the sample size 265 children were allocated to each village/Kebele by Probability Proportional to Size (PPS). After proportionally allocating samples, the study used list of children below the age of five years from the health extension workers of respective villages/Kebeles as a sampling frame. So, systematic random sampling with unique K^th^ value for each village/Kebele was defined and K^th^ interval was used to reach a final study unit.

### 2.3. Data Collection Method

Data on acute respiratory infection were collected through semistructured questionnaire by interviewing mothers or caregivers of under-five children. The questionnaire was adapted from WHO acute respiratory infection in children case management in small hospitals in developing countries [14] and Ethiopian Demographic and Health Survey [15] questions to meet the context of the study area with respect to stove type and housing conditions. Data on indoor air pollution from particulate matter was measured by using China manufactured laser pm 2.5 meter (H0LDPEAK HP-5800D) which is high-technology air quality monitor to measure the pm_2.5_ and pm_10_ using laser scattering principle that ranges from 0 to 999.9 *μ*g/m^3^ rate of detection for pm_2.5_ and from 0 to 1999.9 *μ*g/m^3^ rate of detection for pm_10_ with a resolution of 0.1 *μ*g/m^3^. For the validation of measurement, meteorological factors affecting the concentration of particulate matters like temperature (°C) and relative humidity (RH%) were also monitored and recorded. Written consent was obtained from community/Kebele leaders and each participating household for conducting the survey and monitoring. It was explained to the participants that the survey was solely for research purposes. Data collectors were two public health officers. The data collectors were trained for two days extensively in interviewing techniques, data recording, and approaches to promote health education and child health screening.

#### 2.3.1. Child Health Screening

Health professionals screened children for the prevalence of acute respiratory infections through detail history clerking by interviewing children's mother or primary caregiver by asking whether the child had been presented with sign and symptoms like coughs, accompanied by chest in drawing, shortness of breath, and rapid breathing as described by WHO integrated management of childhood illness in the two weeks preceding the survey interview [16]. Physical examination of chest was done after sitting children in comfortable environment and appropriate disclosure. Starting from inspection of respiratory rate, rhythm, symmetric expansion of chest, and use of accessory muscles for ventilation were assessed. Palpation was done by examining the children posteriorly, placing the thumbs together at the midline at the level of the tenth rib with hands grasping the lateral rib cage; both visual and tactile observations were made during tidal volume breathing and deep forceful inhalation. After inspection and palpation, percussion was made by pressing the distal phalanx of the middle finger firmly on the area to be percussed and raise the second and fourth fingers off the chest surface and then strike the finger in contact with chest wall to determine whether the cavity was filled with air, fluid, or solid. Finally, auscultation of chest using stethoscope was done by crossing children arm anteriorly moving scapula laterally during tidal ventilation, deep forceful inspiration, and forced expiration. Normal and abnormal (wheeze, crackles, and gurgles) were identified using auscultation [17]. With detailed history clerking and physical examinations, children were diagnosed with acute respiratory infections.

### 2.4. Particulate Matter Measurement and Monitoring

Indoor PM (PM_2.5_ and PM_10_) measurements were done in the kitchen, living room, and outdoor. The measurement of indoor particulate matter was done in the following way:

- Approximately 100 cm from the edge of the combustion zone (this distance away from the stove approximates the edge of the active cooking area).
- At a height of sitting position of the cooks or children 1m above the floor (the approximate breathing height of a sitting woman carrying baby or children height).
- At least 150 cm horizontally away from doors and windows, where possible [9].

The outdoor particulate matter was measured at the sampling location of height about 2* *m from the ground level, minimum of 2 m away from obstruction like wall of house, 10 m from trees, 5 meters from the chimney, and 5 meters from the edge of the nearest traffic lane [18].

### 2.5. Operational Definition

#### 2.5.1. Acute Respiratory Infection

It is defined as children with any one or combination of symptoms and signs like cough, shortness of breath, rapid breathing, noisy breathing, and chest in drawing to be differentially diagnosed by clinicians through physical examination of chest (inspection, palpation, percussion, and auscultation).

#### 2.5.2. Biomass Fuel Use

It includes households that use either one or a combination of cooking fuels such as wood, animal dung, charcoal, and kerosene.

#### 2.5.3. Clean Fuel Use

It refers to sole use of electric stoves without mixing other source of fuels.

#### 2.5.4. Indoor Air Pollution

It is by emissions from cooking by using wood, animal dung, charcoal, kerosene, and other cooking fuels.

#### 2.5.5. Ventilation

- Good—when the window to floor area ratio is greater than 20%.
- Fair—when the window to floor area ratio is equal to 10–20%.
- Bad—when the window to floor area ratio is less than 10 [19].

### 2.6. Data Quality Assurance

Semistructured questionnaires that were adapted from WHO and EDHS were first prepared in English and then translated in to Afaan Oromo and Amharic which was translated back into English to ensure consistency of the questions. In order to obtain quality data, before starting actual data collection process, the principal investigator carried the following priority activities. As part of data quality management strategy, two-day training was given for supervisors and data collectors about general concept of ARI, indoor air pollution, aim of the study, data collection tools, process of data collection, and the role of each individual from the starting of data collection to its end. Pretest was done on 5% of population that were not included in the study. The aim of the pretest was to check the sequence of questions, its language comprehension, and duration of time it took to make modification on the questionnaires as necessary according to the findings of the pretest. The pretest found few editorial problems, two more additional questions were added, and repeated questions were removed. Furthermore, to assure the quality of data, the questionnaires were checked for completeness at the end of each data collection day to identify any missing data. Indoor particulate matter and meteorological measurement were taken according to standard methods and procedures. Equipment's in the field measurements were factory calibrated and professionals were manipulating it to ensure validity and accuracy of field data. Triplicates of samples were taken throughout the measurement and the mean was recorded.

### 2.7. Data Analysis and Interpretation

Raw data were entered into EpiData version 3.1. After reviewing and checking the data at different levels of the EpiData software, the data were exported to SPSS version 23 statistical software for analysis. Consistency and completeness of data were checked using frequency and 2 by 2 tables. Descriptive statistics such as frequency distributions and measure of central tendencies were calculated for dependent and independent variables. In addition, normality test was done to check the distribution of the data. Bivariate and multivariate analysis using binary logistic regressions was done to determine the presence of a statistically significant association between predictor variables and the acute respiratory infections. Variables that had a *p* value less than 0.25 on bivariate analysis were selected as candidates for multivariable analysis [20]. To control the effect of covariables while determining the presence of association between explanatory variables and ARI, multivariate logistic regressions analyses were used.

Independent samples *t*-test were employed to compare significant mean difference concentration of particulate matter between biomass and clean fuel. Pearson's correlation analysis was done to determine the relationship between concentration of particulate matter and meteorological parameters like temperature and relative humidity. The overall model fitness was measured using the Hosmer & Lemeshow statistic test and the model is a good fit for the data. On top of this, a test of multicollinearity between the predictor variables was undertaken. Finally, the results were presented using tables and figures.

### 2.8. Ethical Consideration

Ethical clearance and approval were obtained from ethical review board of Jimma University, Institute of Health. A support letter was written from the Department of Environmental Health. Formal letter was given to Kebele administrations and health extension workers. During data collection, each respondent was informed about the purpose, scope, and expected outcome of the research and appropriate verbal and written consent was obtained. The study participants were assured about the confidentiality of the data. Children who were diagnosed with acute respiratory infection were referred for treatment to nearby health institution, and linkage of follow up through community health extension workers was created. Households with high particulate matter concentration were informed about health risk of high particulate matter concentration and its reducing strategies were also pointed.

## 3. Results

### 3.1. Social Demographic Characteristics

A total of 265 samples of children in Jimma town were involved in this study. The response rate was 100%. The overall average family size was 5 persons per household. The examinee's sex was 134 (50.6%) male and 131 (49.4%) female. The mean age of under-five children was 33 months. The marital status distribution of children parents was 218 (82.3%) married, 29 (10.9%), divorced, 10 (3.8%) separated, and 8 (3%) were widowed. In terms of children mother's educational background, 34 (12.8%) were unable to read and write, 99 (37.4%) completed primary education, 66 (24.9%) completed secondary education, 37 (14%) were certified with college diploma, and the rest 29 (10.9%) were certified with university degree and above. The majority of households 197 (74.3%) were institution based, followed by 63 (23.8%) rent houses, and the rest 5 (1.9%) were living in owned houses. Regarding the occupational status of the parent, 147 (55.5%) were working their own business, 66 (24.9%) were government employees, 50 (18.9%) were private company employees, and 2 (0.8%) were NGO employees.

### 3.2. Household Energy Source and Kitchen Characteristics

**Figure 1:**
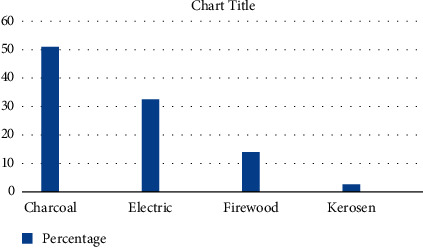
Source of energy distribution of households in Jimma town, Ethiopia, May, 2020.

**Figure 2:**
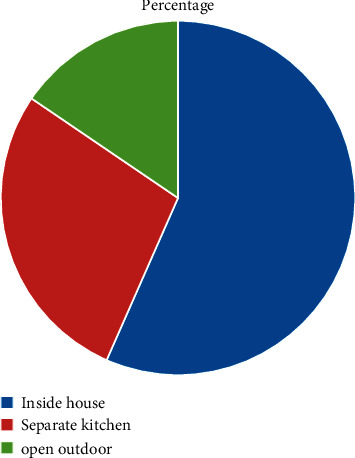
Cooking place distribution of households in Jimma town, Ethiopia, May, 2020.

The majority of the households 135 (50.9%) were using charcoal stove, followed by electric stove 86 (32.5%), three-stone pit stove 37 (14%), and kerosene stove 7 (2.6%). Regarding the cooking place, the majority of the households 150 (56.6) were cooking inside the house without partition of kitchen, 74 (27.9%) were cooking in separate kitchen, and the rest 41 (15.5%) of the households were cooking in the outdoor. Only 21 (7.9%) kitchens were installed with kitchen vent and the rest 244 (92.1%) lack kitchen vent installation. The flooring material of households were dominantly made up of vinyl/asphalt strip 179 (67.5%) and the rest 86 (32.5%) were earth. The dominant roofing material was corrugated iron sheets 263 (99.2%) (Figures 1 and 2).

### 3.3. Prevalence of Acute Respiratory Infections (ARI)

The overall prevalence observed during the two weeks' period was 43 cases, i.e., 16.2% (95% CI: 11.2–19.4) of surveyed children were found to have had ARI. Distribution among gender showed that 24 cases (55.8%) were female and 19 cases (44.2%) were male children. The distribution by age group was found to be 26.4% for those aged less than one year, 23.9% for one year, 20.4% for two years, 17.5% for three years, and 11% for four years old children. Distribution of acute respiratory infections were 35 cases (81.3%) pneumonia, 5 cases (11.6%) bronchiolitis, and 3 cases (6.9%) asthma (recurrent wheeze) **(**Table 1**).**

### 3.4. Association between the Prevalence of Acute Respiratory Infections and Biomass Fuel

A total of 179 (67.5%) households in the study area were primarily dependent on biomass fuel as their major source of energy for household consumption. About 135 households (50.9%) were using charcoal as their primary source of energy, followed by firewood among 37 (14%) households and kerosene among 7 (2.6%) households. Association analysis was done between acute respiratory infections and biomass fuel using Pearson chi-square test and it found statistically significant correlation existing between the two (*x*^2^ = 25.41, *p*=.000).

Bivariate logistic regression was used to estimate the relative association of biomass fuel use and ARI. Result from bivariate logistic regression showed that children living in households that use biomass fuel were five times more likely to develop acute respiratory infections than their counterparts (OR: 5.44; 95% CI: 3.24, 12.45). The association was found statistically significant and able to fit the next model for final analysis by multivariate logistic regression. Results from multivariate logistic regression showed that children living in households that use biomass fuel were four times at increased risk of developing ARI than their counterparts (AOR: 4.348; 95% CI: 1.632, 11.580).

### 3.5. Factors Associated with Acute Respiratory Infection

In the association analysis, using Pearson chi-square test, 23 variables were separately analyzed whether they are associated with ARI prevalence among under-five children, and among them 8 variables were found to be statistically significant at 0.05 and 0.001 levels **(**Tables 2 and 3).

After conducting bivariate logistic regression, from eight lists of explanatory variables that had shown association with acute respiratory infections, only the above four variables were found statistically significant and able to fit the next model for final analysis by multivariate logistic regression. In the multivariate logistic regression, size of household and separate kitchen were found significantly associated with the prevalence of ARI. The size of household was significantly associated with the prevalence of ARI. Under-five children living in households that have a family size of six and greater had odds of 1.7 increased risk of developing acute respiratory infections (ARI) than their counterparts (AOR: 1.7; 95% CI: 1.299, 2.212). The other variable which was significantly associated with acute respiratory infections was separate kitchen. Children living in households in which there were no separate kitchen were four times at increased risk of developing ARI than children living in households which have separate kitchen (AOR: 4.591; 95% CI: 1.849, 11.402) **(**Table 4).

### 3.6. Concentration of Indoor Particulate Matter

The peak average cooking hour concentration of PM_10_ was statistically higher in kitchen than in living room (*t* = 4.509, *p*=.000). On contrary, there was no significant difference in concentration of PM_2.5_ in the kitchen and living room (*p* > 0.05). The mean cooking hour concentrations of PM_2.5_ for households using biomass fuel and clean fuel were 344.03 ± 21.7 and 60.42 ± 7.6, respectively, whereas the mean cooking hour concentrations of PM_10_ for households using biomass fuel and clean fuel were 752.8 ± 52.3 and 143 ± 30.4, respectively. The peak hour concentrations of both PM_2.5_ and PM_10_ were a statistically higher among households using biomass fuel than households that rely on clean fuel (*t* = 9.345, *p*=.000 and *t* = 8.12, *p*=.000) respectively. Correlation analysis of this study showed that the concentration of both PM_2.5_ and PM_10_ was positively correlated with the prevalence of acute respiratory infections (*r* = 0.525, *p*=0.000 and *r* = 0.451, *p*=0.000), respectively.

Regression analysis showed that the concentration of PM_2.5_ was 20 times higher in households where parents were smoking cigarette (AOR: 20.224; 95% CI: 1.72, 12.58) than households where no family members were smoking.

### 3.7. Correlations of PM with Meteorological Parameters

The correlation between the particulate matter concentration, temperature, and relative humidity were evaluated. Based on the results of Pierson correlation, there was no statistically significant correlation between PM2.5 and meteorological parameters. A positive significant correlation was observed between PM10 and temperature (*r* = 0.45, *p*=0.04). Moreover, the concentration of PM10 was negatively correlated with relative humidity (*r* = −0.62, *p*=0.001).

## 4. Discussion

Biomass fuel was the major form of energy supply for household consumption in the study area. 67.5% of households in the study area were primarily dependent on biomass fuel, with the largest share belonging to charcoal followed by firewood. This result is consistent with study done in Wolaita Sodo, Southern Ethiopia, that showed 66% of households were dependent on biomass fuel in the form of charcoal, firewood, and kerosene for household cooking purposes [6]. The result of the study was lower than that of the Ethiopian Demographic And Health Survey that found ninety-three percent of households in Ethiopia use some type of solid fuel for cooking, with virtually all of these households using wood [15]. This variance might be the result of urban nature of the study area when compared to the EDHS study area that comprises both urban and rural corners of the country. Despite the difference in consumption of biomass fuel in different areas in the country, the biomass fuel coverage in the study area is high and responsible for increased amount of household air pollution. The implication of high biomass usage might be related with its easy accessibility and lower cost when compared with the cleaner fuel [21]. The result of this study indicated that the two weeks' prevalence of ARI among children under five years was 16.2%. This study was comparable with a study conducted in Shebedino, Southern Ethiopia, 21% [12]. It was slightly lower than a study done in slum area of Addis Ababa, which showed that 23.9% of under-five children were suffering from ARI [10]. It was higher than the national demographic and health survey that found seven percent of children under the age of 5 had symptoms of ARI in the 2 weeks before the survey [15]. This variation might be due to difference in host factors like underlying diseases that reduce immunity [22]. Underlying disease condition can easily aggravate the immune system and make children vulnerable to acute respiratory infections.

This study shows strong associations between biomass fuel and prevalence of ARI in children. Children living in households that use biomass fuel were four times at increased risk of developing ARI than households that uses clean fuels. It was in line with a study conducted in Wolaita Sodo [6] that reveal children living in households that are reliant on biomass fuels were about four times more likely to suffer from an ARI compared to children living in homes reliant on clean fuel. The result is also comparable with a study done in Addis Ababa, Ethiopia, that showed children living in a house that uses biomass fuel for cooking and heating were three times more likely to develop ARI [10]. It was also consistent with study in Ibadan, Nigeria [5], where the use of biomass fuel was four times more likely to increase the risk of developing ARI than households that were dependent on clean fuels. This might be due to the process of burning biomass fuels in simple stoves with incomplete combustion emitting hundreds of harmful chemical substances in the form of gases, aerosols, and suspended droplets that adversely affect the respiratory tract which result in respiratory infections [23]. Improvement in energy sources from biomass fuel that emits hundreds of harmful chemicals to cleaner sources like electricity could halt indoor air pollution and hence decrease childhood respiratory infections. In this study, household family size was associated with ARI prevalence. Under-five children living in households that have a family size of six and greater were twice more likely to develop acute respiratory infections (ARI) than their counterparts. This was consistent with a study conducted in Shebedino that showed the odds of ARI in children were twice higher in households with a family member of more than five than they were in a family member of five or less [12]. It was supported by the study conducted in Enugu, Southeast Nigeria, where children living in a family size of more than 5 siblings were two times more likely to develop ARI [24]. This might be due to overcrowding, which hasten the contact with discharges and droplets that increase transmission of the disease. The implication of large family size was that the crowding of a house affects the health of inhabitants with high chance of transmission of droplets of infective organisms between them [25]. Reducing over crowdedness can help children from exposure to household air pollution and decrease the risk of disease transmission. It can also be due to increased demand of biomass fuel consumption in larger families compared to their counterparts; increased consumption of biomass fuel at domestic level for lighting, heating, and cooking might escalate the concentration of particulate matter in the indoor environment and results in acute respiratory infections. This is mostly due to the fact that clean fuel is an expensive fuel source which is rarely affordable for larger-sized households forcing them to choose cheaper alternatives [26].

The result of this study showed that kitchen type was significantly associated with acute respiratory infections. Children living in households in which there were no kitchen partitions were four times at increased risk of developing ARI than children living in households that have separate kitchen. This result was in agreement with a study conducted in Nigeria in which cooking was done in the same room where the child sleeps and thus the child was found to have a 3-fold greater risk of ARIs than children belonging to households that have its separate kitchen [5]. It was also consistent with the study in Burkina Faso, in which children in household, where the kitchen were inside the living room, were found to be four times more likely to develop ARI than children of their counterparts [27]. This implies that combustion of biomass fuels in internal kitchens that lack partition would lead to the circulation of particulate matters directly from the cooking area to living room which increases the risk of getting ARI [28].

The result of this study showed that there was statistically significant higher particulate matter concentration among households using biomass fuel than clean fuel. It was consistent with the study finding in Sir Lanka that showed houses which used biomass fuel for cooking had significantly higher concentrations of PM (*p* < 0.001) as compared to houses using LPG and electricity [1]. It was also in line with a study conducted in India in which the use of biomass fuel resulted in higher concentrations of indoor particulate matter compared to clean fuel [9]. This implies biomass fuel had low combustion efficacy leading to huge emission of health damaging air pollutants including particulate matter [10]. The finding of this study showed that the concentrations of PM _2.5_ were higher in households where parents were smoking cigarettes than households were no family members were smoking. It was in good agreement with a study done in Scotland which showed that high concentrations of particulate matter were found in homes with a smoker resident who smoked indoors [29]. Cigarette smoke contains 7,357 different chemical compounds; the particles emitted during smoking settle in the indoor environment and increases household air pollution [30].

## 5. Conclusion

Biomass fuel is the dominant energy source used for cooking in the study area. There is a strong association between biomass fuel and prevalence of ARI in under-five children. Family size and kitchen characteristics influence the prevalence of ARI in under-five children. Concentration of particulate matter was higher in the kitchen than living rooms. Higher concentration of particulate matter was associated with households relying on biomass fuel than clean fuel. Based on the findings of the study, the following recommendations are forwarded:

- Households need to improve kitchen environment to reduce household air pollution.
- Parents of under-five children need behavior adjustments such as ensuring children are kept away from smoke and not smoking in the indoor to reduce indoor air pollution and subsequent acute respiratory infections.
- Government needs an effective strategy for improving fuels and cooking technologies to reduce the high biomass usage in the study area.
- Further study is recommended to better understand how interventions on IAP reduction are effective to reduce the health risks among children.

## Figures and Tables

**Table 1:** Prevalence of acute respiratory infections with respect to age, sex, and diagnosis among under-five children in Jimma town, Ethiopia, May, 2020.

Sn. No.	Characters	Categories	Prevalence
1	Age	Less than one year	11 (25.6%)
		One year	10 (23.3%)
		Two years	9 (20.9%)
		Three years	8 (18.6%)
		Four years	5 (11.6%)
		Total	43 (100%)

2	Sex	Male	19 (44.2%)
		Female	24 (55.8%)
		Total	43 (100%)

3	Diagnosis	Pneumonia	35 (81.3%)
		Bronchiolitis	5 (11.6%)
		Asthma (recurrent wheeze)	3 (6.9%)
		Total	43 (100%)

**Table 2:** Test of association of explanatory variables with outcome variable among under-five children in Jimma town, Ethiopia, May, 2020.

No.	Explanatory variables	Pearson's chi-square (*x*^2^)	Significance
1	Family size	50.23	.000
2	Level of education	9.79	.044
3	Type of stove	25.415	.000
4	Habit of carrying baby on back while cooking	12.418	.000
5	House flooring materials	17.316	.000
6	Separate kitchen	42.168	.001
7	Smoker among family	54.368	.000
8	Frequency of smoking	51.935	.000

**Table 3:** Results of bivariate logistic analysis of independent variables among under-five children in Jimma town, Ethiopia, May 2020.

Variables	B	S.E.	Wald	df	sig	Ex(B)
HH size ≥6	2.013	.380	28.019	1	.000	7.489
HH size <6^*∗*^						
Smoking = yes	1.818	.177	105.265	1	.000	6.162
Smoking = no^*∗*^						
Carry baby on back = yes	1.874	.182	106.558	1	.000	6.514
Carry baby on back = no^*∗*^						
Separate kitchen = no	1.618	.233	48.096	1	.000	5.045
Separate kitchen = yes^*∗*^						

^*∗*^Reference category.

**Table 4:** Results of multivariate logistic analysis.

Variables	B	S.E.	Wald	df	sig	Ex(B)	95% CL Ex(B)	
							Lower	Upper
HH size ≥6	.528	.136	15.125	1	.000	1.695	1.299	2.212
HH size <6^*∗*^								
Separate kitchen = no	1.524	.464	10.783	1	.001	4.591	1.849	11.402
Separate kitchen = yes^*∗*^								

^*∗*^Reference category.

## Data Availability

The data used to support the finding of this study are included within the article.
